# The Impact of Race and Physician–Patient Racial Concordance on the Incidence of Inpatient Advance Care Planning

**DOI:** 10.1007/s11606-026-10231-x

**Published:** 2026-03-19

**Authors:** Benjamin Carter, Satveer Kaur-Gill, Megan Murphy, A. James O’Malley, Amber E. Barnato

**Affiliations:** 1 Department of Political Science, University of Alabama, Tuscaloosa, AL USA; 2 Department of Communication Studies, University of Nebraska-Lincoln, Lincoln, NE USA; 3 The Dartmouth Institute for Health Policy and Clinical Practice, Geisel School of Medicine at Dartmouth, Lebanon, NH 03755 USA; 4 Department of Biomedical Data Science, Geisel School of Medicine at Dartmouth, Lebanon, NH USA; 5 Section of Palliative Care, Department of Medicine, Dartmouth Hitchcock Medical Center, Lebanon, NH USA

**Keywords:** advance care planning, racial concordance, serious illness, health disparities, patient–provider communication, end-of-life care, Medicare

## Abstract

**Background:**

Racial disparities in end-of-life care have been well documented, yet little is known about how both patient and provider races, as well as their concordance, influence the likelihood of advance care planning (ACP) discussions during hospitalization.

**Objective:**

To evaluate how patient race, provider race, and patient–provider racial concordance are associated with the occurrence of inpatient ACP conversations.

**Design:**

Retrospective observational cohort study using hierarchical logistic regression.

**Participants:**

Seriously ill Medicare beneficiaries hospitalized between 2016 and 2019, managed by a national physician staffing organization (PSO) across 220 hospitals in 35 US states. The final sample included 390,392 hospitalizations and 2808 providers.

**Main Measures:**

The primary outcome was the occurrence of an ACP conversation, identified using CPT codes 99497 and 99498, assessed from admission through day 10 or discharge. Patient and provider races were categorized as White, Black, Hispanic, or Asian. Models included fixed effects for patient demographics, clinical risk, and hospital characteristics, and random effects for hospital clustering.

**Key Results:**

Asian providers were more likely and Hispanic providers less likely to engage in ACP discussions. Patient–provider racial concordance modestly increased the likelihood of ACP for Black, White, and Hispanic patients, and several cross-race pairings also showed higher engagement. These effects were modest, varied across racial dyads, and occurred in the context of higher than national average inpatient ACP rates under the PSO’s quality improvement initiative.

**Conclusions:**

Provider race and patient–provider concordance each influenced the likelihood of inpatient ACP, though effects were modest and context-dependent. Concordance and certain racial pairings were associated with higher engagement, but disparities persisted across groups, highlighting that broader structural and communication barriers continue to shape inequities in end-of-life care.

**Graphical Abstract:**

**Figure:**
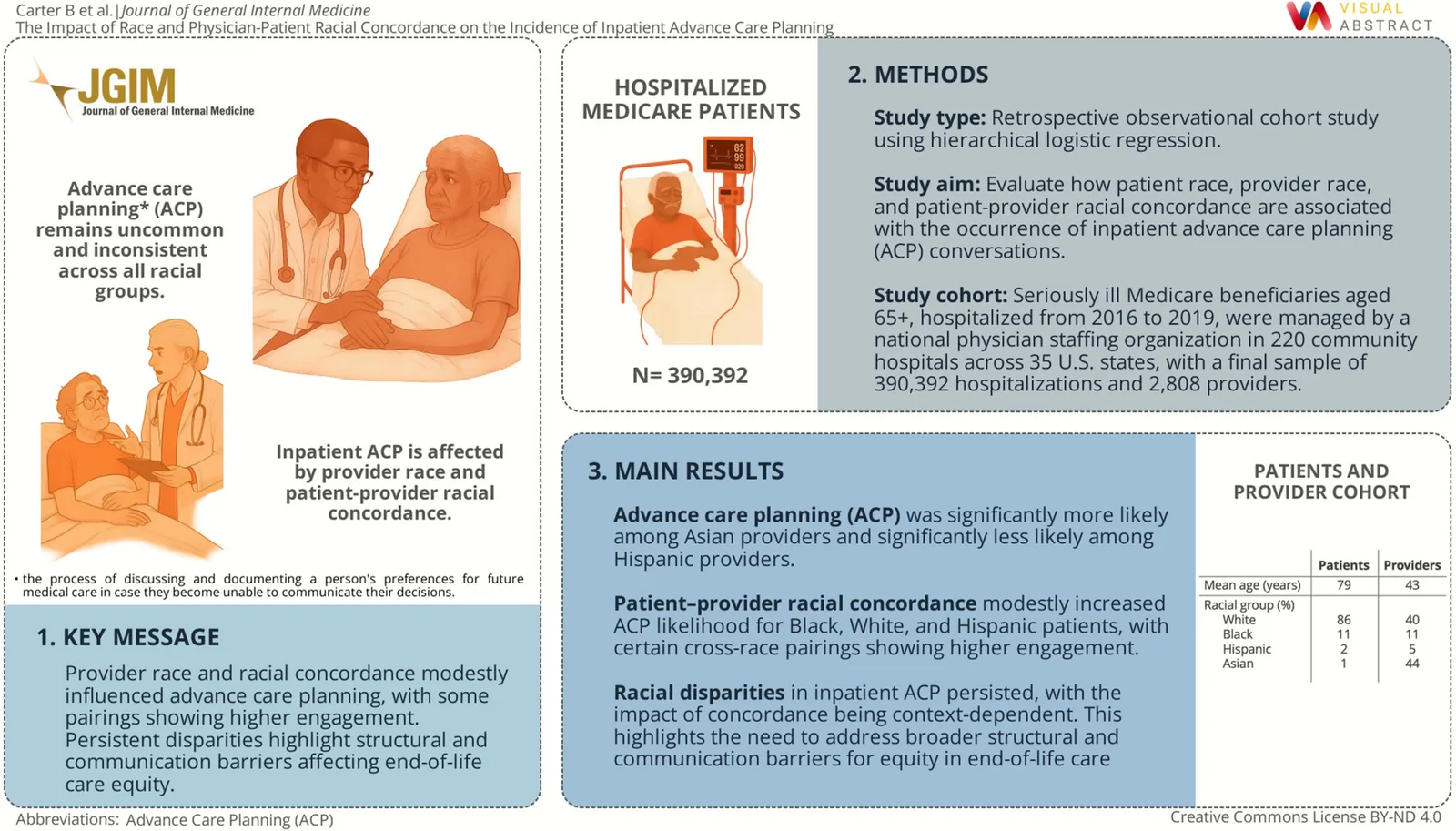

**Supplementary Information:**

The online version contains supplementary material available at https://doi.org/10.1007/s11606-026-10231-x.

## INTRODUCTION

Advance care planning (ACP) conversations are discussions between patients and providers about treatment goals and preferences near the end of life (EOL). ACP improves adherence to patients’ wishes^1^ and is associated with greater hospice and palliative care use and fewer burdensome hospitalizations.^2,3^ However, Black, Asian, and Hispanic patients are less likely than White patients to have ACP conversations, particularly among those with fewer socioeconomic resources, contributing to disparities in EOL care.^4^ In one study of 7177 Health and Retirement Study decedents (2000–2012), 51.7% of White, 18% of Hispanic, and 15% of Black persons had advance directives.^5^ In a 2019 California survey of 1635 adults, 47% of White and 33% of Black respondents reported written advance directives.^6^

Black, Hispanic, and Asian patients face distinct barriers to advance care planning (ACP). Black patients may avoid ACP because of religious beliefs that God determines death’s timing and nature, while Hispanic patients may avoid planning to spare their families.^7^ Hispanic patients also show greater uncertainty about treatment preferences, especially with limited literacy,^8^ and Black patients experience more decisional conflict, which clearer information can reduce.^9^ Asian patients often prefer culturally specific or family-centered approaches^10^ and most often cite not wanting to think about dying as their reason for lacking a living will or healthcare proxy. Both Hispanic and Asian patients frequently report not knowing how to begin ACP discussions.^4^

Qualitative research shows that non-Black physicians often express uncertainty when communicating with Black patients about ACP, leading to anxiety-provoking interactions.^11^ In advanced cancer care, Black patients reported feeling dismissed more often than White patients in racially discordant encounters and adapted their communication strategies accordingly.^12^

Patient trust—the belief that a provider acts in the patient’s best interest—is central to effective communication.^13,14^ Racial concordance, in which patient and provider share a racial or ethnic background, may strengthen that trust and communication, although evidence is mixed.^15^

Physician–patient racial concordance may improve communication and outcomes by fostering trust, shared cultural understanding, and comfort. Experimental studies show that patients in race-concordant dyads feel greater similarity (especially when providers use brief self-disclosure to build rapport), trust, satisfaction, and willingness to continue care or recommend the provider.^16,17^ Broader reviews note that although effects are mixed, concordance can enhance patient experience through cultural comfort, shared language, and fewer relational barriers.^15^ These mechanisms may reduce anxiety, increase respect, and support openness in sensitive ACP discussions.

Members of minoritized racial and ethnic groups often report lower generalized trust, shaped by structural racism and socioeconomic disadvantage.^18–20^ For instance, 70% of Black respondents trusted other Black individuals but only 23% trusted White individuals.^21^ Black patients also report healthcare system distrust stemming from discrimination and unequal treatment.^22–26^

Physician–patient racial concordance has been linked to outcomes such as shorter emergency department wait times and lower healthcare spending^27^, reduced Black infant mortality in complex births^28^, better communication and satisfaction^29,30^, and fewer reports of poor communication in discordant dyads^31^, although some studies show mixed or null effects.^32^ Strained communication in racially discordant encounters may reduce ACP conversations. Although prior studies examined patient race and ACP^33–36^, this is the first to assess how patient–provider racial concordance influences ACP.

ACP conversations require mutual agreement to engage in sensitive discussions about EOL goals of care. Because people often trust those of the same race more, racial concordance could foster the rapport needed for ACP. In this study, we hypothesized that racial concordance would increase the likelihood of ACP conversations between hospitalized patients and providers without a prior relationship. We investigated this possibility by examining whether patient and provider races predicted the likelihood of ACP conversations for seriously ill patients.

## METHODS

### Approach

We examined how patient and provider races and racial concordance influenced ACP, including individuals identified as Asian, Black, Hispanic, or White. Because of inconsistent racial classification by the physician services organization (PSO) and limited validity of CMS categories beyond these groups^37^, we excluded those listed as Native American, Alaska Native, Pacific Islander, multiracial, or unknown race. Patients were assigned to providers based on weekly hospitalist schedules, creating as-if random assignment that enhances causal inference on the effects of race and concordance on ACP conversations.

### Data Source: Providers

We obtained provider data from our partner PSO. The PSO is a for-profit company supplying acute care clinicians to community hospitals (100–500 beds) in over 40 US states. We selected providers employed between 2016 and 2019, and captured data including their national provider identifier (NPI), race, ethnicity, gender, age, provider type (MD, DO, APP), and education. Race and ethnicity were self-reported by physicians.

Given evidence that internationally trained physicians may face greater communication barriers and patient mistrust^38–40^, we identified International Medical Graduates (IMGs) using provider education data. We classified training location using natural language processing and manual review.

In 2016, the PSO launched a quality improvement program encouraging ACP documentation through billing education, small financial incentives, and priming via the “surprise question” (“Would you be surprised if this patient died in the next year?”). We focused on providers who participated in this initiative, as their ACP performance is measurably higher than usual care.^41^

### Data Source: Patients

Patient data were obtained from Centers for Medicare and Medicaid Services (CMS) Part A and B Medicare fee-for-service files, including demographic, diagnostic, and billing information. Eligible hospital stays were identified by linking MedPAR records to inpatient Part B claims with matching provider NPIs and inpatient place-of-service codes. We excluded claims not billed under the PSO and included only hospitals contributing ≥ 1000 stays during the study period.

The sample was restricted to patients aged 65 and older with serious illness (per Kelley et al., 2021). Covariates included age, sex, race, ethnicity, and Charlson comorbidities. Race and ethnicity were self-reported at Medicare enrollment; missing data were imputed using the Research Triangle Institute algorithm and reported in CMS claims.^42^ To assess illness severity, we used a 1-year lookback to estimate predicted mortality risk at admission via the van Walraven logistic model (detailed in^41^). Predictors included demographics, admitting diagnosis, comorbidities, ICU use, prior ED visits, home oxygen, recent readmissions, nursing care, and serious illness indicators (Appendix II). The model was trained on a broader PSO dataset to ensure exogeneity.

Provider responses to the “surprise question” served as a second illness severity measure; a “No” response triggered an EOL flag on the patient census list, priming the provider for ACP. We included only patients seen by PSO-affiliated providers at PSO hospitals from 2016 to 2019. Daily billing data linked each patient–provider dyad by NPI and associated it with provider race.

### Advance Care Planning

ACP was identified using CMS billing codes 99497 (initial 16–30 min) and 99498 (each additional 30 min). We tracked ACP billing for each hospital day and censored observations after the first billed ACP conversation, hospital discharge, or day 10—whichever occurred first. This approach emphasizes the first, most consequential ACP encounter and aligns with the PSO’s admission-level performance targets. Subsequent ACP conversations and hospital stays exceeding 10 days were excluded, as they may reflect unmeasured clinical or psychosocial variation.

### Statistical Modeling

We used four race categories: White, Asian, Hispanic, and Black. As described above, we conceptualized race as a social construct shaping trust and rapport in racially concordant versus discordant encounters. We examined whether ACP likelihood was associated additively or interactively with patient and provider race while accounting for other covariates. To test our hypotheses, we estimated models including main effects and interaction terms representing three levels of concordance specificity: (1) concordant vs. non-concordant, (2) race-specific concordance with all non-concordant pairs combined, and (3) fully unstructured, allowing each patient–provider racial pairing its own effect.

We estimated four mixed-effects logistic regression models with random hospital effects to account for clustering of patients within hospitals and the repeated measurements generated from multiple encounters for the same patient, modeling all encounters until the first billed ACP conversation or up to 10 hospital days.

- Model 1: Main effects of patient and provider races and all patient/provider covariates.
- Model 2: Model 1 plus a binary indicator for concordance (1 = concordant).
- Model 3: Model 2 plus allowing distinct concordance effects for each race.
- Model 4: Model 1 plus a fully specified interaction of patient and provider races, allowing unique effects for each concordant and discordant combination.

We also attempted to include provider random effects but model convergence failed; thus, the hospital random effect captures both patient and physician clustering. Models adjusted for patient and provider sex and age, Charlson comorbidities, the provider’s “surprise question” response, and IMG status. These covariates are denoted as *PatientChar* and *PhysChar* in Appendix I, which provides full equations and specification tests.

To compare fit across models, we used type III analysis of variance with chi-square tests; no significant differences emerged (all *p* > 0.1).

## RESULTS

### Sample Characteristics

The dataset included 1,234,169 encounters during 390,392 hospital stays managed by 2808 physicians across 220 hospitals. The mean patient age was 79 years and the mean provider age was 43 years. Most patients were White (86%), followed by Black (11%), Hispanic (2%), and Asian (1%). Among providers, 40% were White, 44% Asian, 11% Black, and 5% Hispanic. Most (87%) were physicians rather than advanced practice providers, and 54% were international medical graduates (IMGs) (see Table 1). Approximately one-third (35%) of encounters were racially concordant. Table 2 displays the number of encounters by patient and provider races.

**Table 1:** Cohort Characteristics

	Patient stays	Providers
*N*	390,392	2808
Age *M* (SD)	79 (9)	43 (9)
Female *N* (%)	215,953 (55%)	1149 (42%)
Race *N* (%)		
* White*	333,989 (86%)	1119 (40%)
* Black*	42,473 (11%)	306 (11%)
* Hispanic*	8172 (2%)	153 (5%)
* Asian*	5758 (1%)	1230 (44%)
Mortality model score *M* (SD)		
* White*	0.44 (0.18)	
* Black*	0.43 (0.19)	
* Hispanic*	0.43 (0.18)	
* Asian*	0.43 (0.19)	
Charlson comorbidity score *M* (SD)		
* White*	10.9 (4.5)	
* Black*	12.3 (4.5)	
* Hispanic*	12.0 (4.7)	
* Asian*	11.3 (4.6)	
International medical graduate *N* (%)		1470 (54%)
Year of admission *N* (%)		
* 2016*	54,987 (14%)	
* 2017*	112,325 (29%)	
* 2018*	119,635 (31%)	
* 2019*	103,445 (27%)	
Provider type *N* (%)		
* MD or DO*		2377 (87%)
* Mid-level, advance practice provider*		365 (13%)

**Table 2:** Number of Encounters by Patient and Provider Races

	White Patient	Asian Patient	Hispanic Patient	Black Patient	All Patients
White Provider	*395,323* *87.76%*	5493 1.22%	8118 1.80%	41,511 9.22%	450,445 100%
Asian Provider	510,266 84.66%	*11,108* *1.84%*	12,734 2.11%	68,634 11.39%	602,742 100%
Hispanic Provider	49,354 83.96%	587 1.00%	*2692* *4.58%*	6166 10.49%	58,799 100%
Black Provider	97,862 80.08%	1738 1.42%	2194 1.80%	*20,389* *16.69%*	122,183 100%
All Providers	1,052,805 85.34%	18,926 1.53%	25,738 2.09%	136,700 11.08%	1,234,169 100%

Italicized cells indicate concordant encounters

### Unadjusted ACP Rates

Among patient stays, 19.4% included at least one ACP bill, with no significant variation by patient race: 20.5% for Asian, 19.3% for Black, 20.4% for Hispanic, and 19.4% for White patients (*χ*^2^(3, *N* = 220,780) = 5.68, *p* = 0.128). These rates were substantially higher than national benchmarks (< 2% in non-PSO hospitals) due to the PSO’s quality improvement initiative.^41^ At the encounter level—the unit of primary analysis—4.4% of patient–provider days included an ACP bill, varying significantly by race: 4.5% for Asian, 4.1% for Black, 4.6% for Hispanic, and 4.4% for White patients (*χ*^2^(3, *N* = 1,234,169) = 32.91, *p* < 0.001). ACP rates in racially concordant dyads (4–5%) were comparable to overall encounter rates.

### Adjusted Analysis

Table 3 presents results from four mixed-effects logistic regression models examining how physician and patient races—individually and interactively—predicted the likelihood of an ACP conversation while adjusting for potential confounders. In all models, Asian providers had significantly higher odds of ACP than White providers (Model 4: OR = 1.05, 95% CI 1.020–1.086, *p* < 0.01). Across models, ACP likelihood increased when patients were older, female, or sicker, and when providers were younger or male. As expected, the odds of ACP more than doubled when providers indicated they would not be surprised if the patient died within a year (Model 4: OR = 2.23, 95% CI 2.166–2.286, *p* < 0.001), reflecting the PSO screening tool’s role in prompting ACP discussions.

**Table 3:** Logistic Regressions where Physician and Patient Race are Used to Predict Odds Ratios for ACP Conversations

	**Model 1**		**Model 2**		**Model 3**		**Model 4**	
Patient race (White comparison)								
* Asian*	0.93	(0.042)	0.90*	(0.042)	1.04	(0.076)	0.93	(0.076)
* Hispanic*	1.10*	(0.044)	1.13**	(0.046)	1.14**	(0.050)	0.87	(0.066)
* Black*	1.03	(0.021)	1.04*	(0.022)	1.06*	(0.027)	0.94	(0.035)
Provider race (White comparison)								
* Asian*	1.07***	(0.016)	1.16***	(0.030)	1.21***	(0.046)	1.05**	(0.017)
* Hispanic*	0.98	(0.032)	1.06	(0.041)	1.10*	(0.053)	0.98	(0.034)
* Black*	1.01	(0.023)	1.08**	(0.031)	1.13**	(0.048)	0.98	(0.024)
Race interactions								
* Concordance*			1.09***	(0.027)				
* White concordance*					1.15***	(0.045)		
* Asian concordance*					0.89	(0.082)	1.00	(0.099)
* Hispanic concordance*					1.10	(0.164)	1.41*	(0.230)
* Black concordance*					1.08	(0.063)	1.21**	(0.078)
* Asian (patient) × Hispanic (provider)*							0.71	(0.264)
* Asian (patient) × Black (provider)*							1.11	(0.176)
* Hispanic (patient) × Asian (provider)*							1.40***	(0.129)
* Hispanic (patient) × Black (provider)*							1.37*	(0.206)
* Black (patient) × Asian (provider)*							1.13**	(0.050)
* Black (patient) × Hispanic (provider)*							0.88	(0.103)
Patient age	1.01***	(0.001)	1.01***	(0.001)	1.01***	(0.001)	1.01***	(0.001)
Provider age	0.99***	(0.001)	0.99***	(0.001)	0.99***	(0.001)	0.99***	(0.001)
Patient female	1.06***	(0.013)	1.07***	(0.013)	1.06***	(0.013)	1.07***	(0.013)
Provider female	0.88***	(0.011)	0.88***	(0.115)	0.88***	(0.012)	0.88***	(0.012)
Mortality model score	2.64***	(0.104)	2.64***	(0.104)	2.64***	(0.104)	2.64***	(0.104)
Surprise question	2.23***	(0.030)	2.23***	(0.030)	2.23***	(0.030)	2.23***	(0.030)
International medical graduate	0.94***	(0.015)	0.94***	(0.015)	0.94***	(0.015)	0.94***	(0.015)
Number of encounters	827,213		827,213		827,213		1,214,907	
Model fit (AIC)	409,343.2		409,320.0		409,311.9		409,297.9	

Models control for Charlson comorbidities and include random effects for hospitals. Model 1 includes no interaction terms, Model 2 adds a binary term for racial concordance, Model 3 includes binary terms for race-specific concordance, and Model 4 fully interacts patient and provider races with White patients and providers as the comparison group. The number of encounters was reduced from 1,214,907 to 827,213 in the models because of missing Surprise Question data. Standard errors are in parentheses. *p < 0.05; **p < 0.01; ***p < 0.001

Patient–provider racial concordance was a significant predictor of ACP across models. Model 2 used a binary variable and found that concordance was associated with 10% higher odds of ACP (OR = 1.09, 95% CI 1.041–1.148, *p* < 0.001). In race-specific analyses, Black patients who saw Black providers had 21% higher odds in Model 4 (OR = 1.21, 95% CI 1.068–1.375, *p* < 0.01), but this association was not significant in Model 3 (OR = 1.08, 95% CI 0.964–1.210, *p* = 0.186). White patients who saw White providers had 15% higher odds in Model 3 (OR = 1.15, 95% CI 1.066–1.243, *p* < 0.001) and served as the comparison group in Model 4. Hispanic patients who saw Hispanic providers had 41% higher odds in Model 4 (OR = 1.41, 95% CI 1.028–1.940, *p* < 0.05), but this was not significant in Model 3 (OR = 1.10, 95% CI 0.821–1.475, *p* = 0.521). Concordant Asian encounters were not associated with ACP in either Model 3 (OR = 0.89, 95% CI 0.744–1.067, *p* = 0.209) or Model 4 (OR = 1.00, 95% CI 0.823–1.213, *p* = 0.992).

The full interaction in Model 4 revealed nuanced effects. Hispanic patients had higher odds of ACP when seen by Asian (OR = 1.40, 95% CI 1.170–1.678, *p* < 0.001) or Black (OR = 1.36, 95% CI 1.016–1.835, *p* < 0.05) providers compared with White providers. Black patients had 13% higher odds when seen by Asian providers (OR = 1.13, 95% CI 1.030–1.230, *p* < 0.01) compared with White providers.

**Figure. 1:**
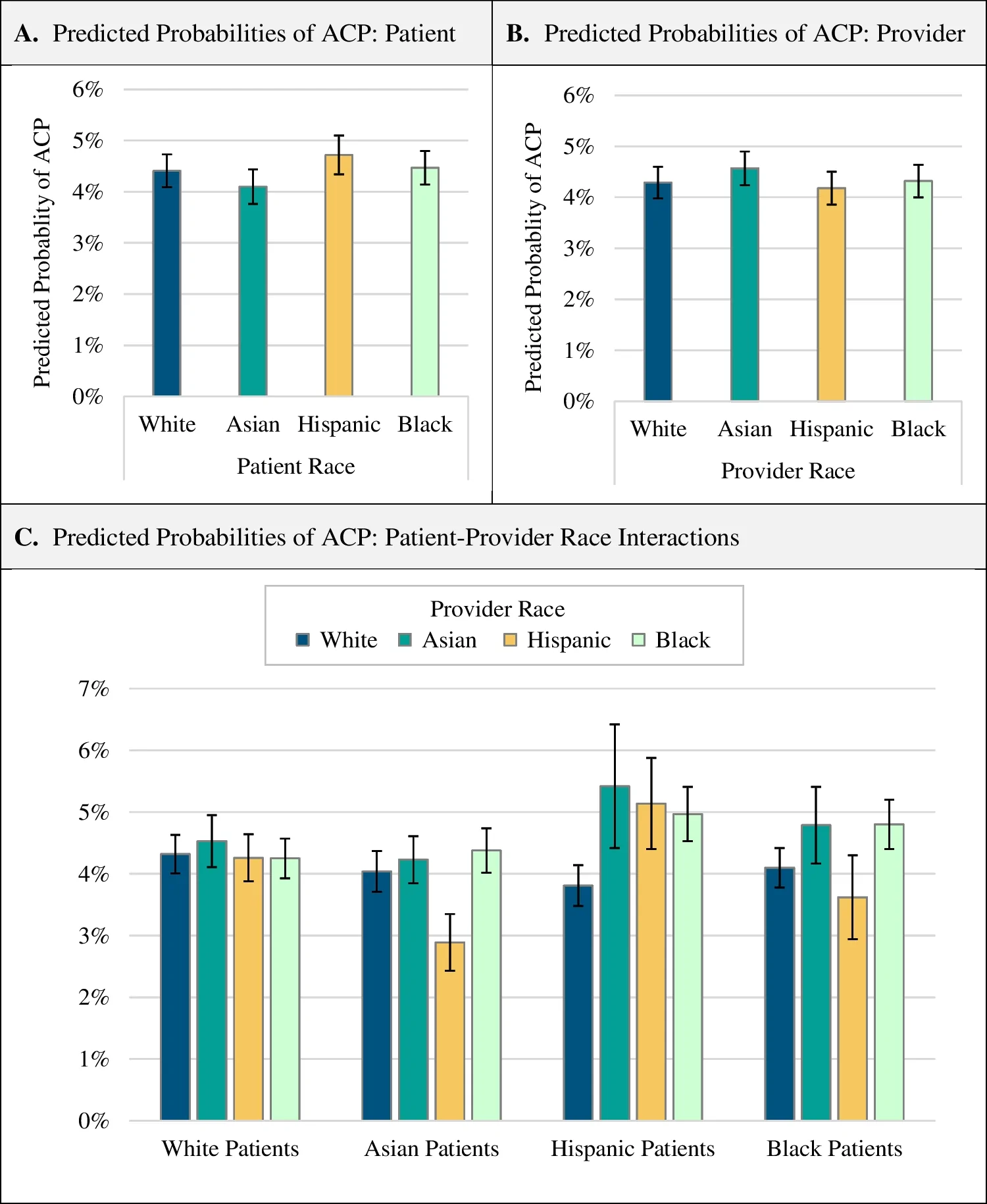
Predicted probabilities of ACP conversations by patient and provider races. Note: Error bars are standard errors

Figure 1 and Table 4 show predicted probabilities of ACP by patient and provider races. In Panel A, probabilities vary slightly across patient race: White 4.41%, Asian 4.10%, Hispanic 4.72%, and Black 4.47%. Panel B shows Asian providers had a slightly higher probability (4.57%) of ACP than White (4.29%) or Black (4.32%) providers, while Hispanic providers had a lower probability (4.18%).

**Table 4:** Predicted Probabilities of ACP Conversations by Patient and Provider Races

	White Patient	Asian Patient	Hispanic Patient	Black Patient	All Patients
White Provider	*4.32%* *(0.31%)*	4.04% (0.42%)	3.81% (0.38%)	4.10% (0.32%)	4.29% (0.31%)
Asian Provider	4.53% (0.33%)	*4.23%* *(0.38%)*	5.42% (0.46%)	4.79% (0.36%)	4.57% (0.33%)
Hispanic Provider	4.26% (0.33%)	2.89% (1.00%)	*5.14%* *(0.74%)*	3.62% (0.44%)	4.18% (0.32%)
Black Provider	4.25% (0.32%)	4.38% (0.62%)	4.97% (0.68%)	*4.80%* *(0.40%)*	4.32% (0.32%)
All Providers	4.41% (0.32%)	4.10% (0.34%)	4.72% (0.38%)	4.47% (0.33%)	

Standard errors are in parentheses. Italicized cells indicate concordant encounters

Panel C displays patient–provider combinations. For White patients, ACP rates vary minimally by provider race (4.04–4.53%, overlapping error bars). Among Asian patients, ACP is lowest with Hispanic providers (2.89%). Hispanic patients had higher ACP rates with concordant Hispanic (5.14%) providers than with White providers (3.81%). Similarly, Black patients had higher rates with Black (4.80%) providers than with White providers (4.10%).

### Sensitivity Analyses

White patients comprised 86% of our analytic cohort. As a sensitivity analysis, we re-estimated models on a random subsample balanced across patient racial groups (*n* = 3000 patients per group; Appendix III) to assess robustness. In the full sample, Hispanic patients appeared to have higher odds of ACP than White patients. In the balanced models, both Hispanic and Asian patients were significantly less likely than White patients to complete ACP, and Black patient effects remained inconsistent. Provider race effects in the subsample were diminished, and concordance was not significant. The balanced subsample included only 3% of the full analytic cohort, so these results should be interpreted cautiously.

## DISCUSSION

In a nationwide sample of acute care providers treating seriously ill, hospitalized older adults, we found that patient–provider racial concordance was associated with increased likelihood of ACP billing across models and groups, particularly among White, Black, and Hispanic patients, though some models showed null effects. We also identified significant nuanced interactions between patient and provider races on ACP. Though prior studies^33,35,43,44^ highlight persistent disparities in ACP among Black and Hispanic patients, our findings indicate that a concerted quality improvement initiative as well as a diverse provider group that increased racial concordances may have helped narrow these gaps. While mechanisms remain uncertain, prior research suggests that culturally responsive communication^4,7^ and greater trust in concordant encounters may contribute.

Unlike prior research focused mainly on Black–White concordance, this study examined multiple racial and ethnic pairings. Although results varied across dyads, the overall pattern underscores the context-dependent role of racial identity in shaping ACP. Prior studies found that racial concordance can improve communication quality, satisfaction, and trust in primary care^29,45,46^ and, in oncology and end-of-life settings, reduce mistrust and increase willingness to plan,^47,48^ while other studies have found null effects.^49,50^ We find that Black, White, and Hispanic racial concordance, as well as particular racial dyads (e.g., Black provider, Hispanic patient), can facilitate ACP.

The higher odds we observe for Black and Hispanic patients may reflect the PSO’s ACP quality improvement initiative.^41^ For Black patients, this could indicate improved communication, consistent with evidence that engagement rises when trust barriers are reduced.^45,51^ For Hispanic patients, lower ACP rates are linked typically to language and family norms.^52,53^ Notably, Black, Hispanic, and Asian patients had *lower* odds of ACP than White patients in sensitivity models balanced on patient race.

This study has several limitations. First, ACP billing codes likely underestimate true practice: a chart review found that only 57% of documented ACP conversations were billed, indicating high specificity but low sensitivity.^54^ Although billing requires at least 15 min of face-to-face discussion, claims data cannot capture discussion quality or depth. Future research should examine how concordance influences the substance and outcomes of these conversations.

Second, our analysis was limited to inpatient ACP in community hospitals (100–500 beds), which may differ from outpatient or larger systems. Third, the PSO’s ACP-focused quality improvement initiative and a workforce with more Asian and internationally trained providers than the national average may limit generalizability. Fourth, although claims data cannot identify declined ACP conversations, a chart review of 434 stays in the parent study found no such documentation. Fifth, we lacked patient education data, which may influence both ACP and racial concordance.

Another limitation is the imbalance in provider race, with White and Asian clinicians comprising most of the sample. This uneven distribution increased power to detect effects involving White providers but limited power for comparisons involving Black and Hispanic providers. Consequently, disparities in White provider encounters should be interpreted with greater confidence than the null or inconsistent findings among Hispanic or Black provider groups.

Racial categories in this study reflect administrative classifications that may obscure important heterogeneity—especially within the broad “Asian” label. Future research should refine how racial and ethnic identity is measured in studies of concordance. Finally, there is no clear definition of “clinically significant” differences in ACP rates. In the pragmatic clinical trial following this study, we powered to detect a 1% absolute increase in ACP.^50^ While improving goal-concordant treatment for even one patient is laudable, such differences may have limited population-level impact.^54^

## CONCLUSION

ACP aligns patients’ EOL treatment with their goals and is a critical component of patient-centered care. We found that provider race is a significant predictor of ACP conversations, with Asian providers more likely and Hispanic providers less likely to engage in ACP discussions. We also found that patient–provider racial concordance was associated with an increased likelihood of ACP conversations for Black, White, and Hispanic patients, suggesting that racial concordance can be an important factor in promoting equity in goal-concordant EOL care.

## Supplementary Information

Below is the link to the electronic supplementary material.

ESM 1 (25.7 KB DOCX)

## Data Availability

The data used in this study are not publicly available due to CMS data use restrictions and contractual agreements with the physician staffing organization that prohibit external sharing.
