# Supplementary material for: The Impact of Race and Physician–Patient Racial Concordance on the Incidence of Inpatient Advance Care Planning
Source: J Gen Intern Med. 2026 Mar 19;41(12):3383–91. doi: 10.1007/s11606-026-10231-x (PMC13055204; doi:10.1007/s11606-026-10231-x)
Supplement: Supplementary file 1 [file 11606_2026_10231_MOESM1_ESM.docx]

**Appendix I**

Analytic Model
Let $PatientRace_{hi}$ and $ProviderRace_{hi}$ be categorical random variables taking values from the set $Race=\{Black,White,Hispanic,Other\}$ where the subscript $h$ denotes hospital and the subscript $i$ denotes patient within hospital. In addition, let $K$ and $L$ denote elements of the set $Race$, defined above. The three interaction variables are defined for observation $(h,i)$ using the ensemble of binary variables given by:

$ConcordantRace_{hi}=1$ if $PatientRace_{hi}=ProviderRace_{ih}$ and 0 otherwise

$ConcordantRaceK_{hi}=1$ if $PatientRace_{hi}=ProviderRace_{hi}=K$ and 0 otherwise

$CombinationRace{KL}_{hi}=1$ if $PatientRace_{hi}=K$ and $ProviderRace_{hi}=L$ and 0 otherwise

Because $Race$ has four levels, it follows that there is a single $ConcordantRace_{hi}$ variable, 4 $ConcordantRaceK_{hi}$ variables, and 16 $CombinationRace{KL}_{hi}$ variables. However, to allow easy interpretation of the coefficients of the interaction variables, both $PatientRace_{hi}$ and $ProviderRace_{hi}$ are included in the model meaning that the coefficients of only 3 of the $ConcordantRaceK_{hi}$ variables can be estimated and the coefficients of only 9 of the $CombinationRace{KL}_{hi}$ variables can be estimated.

The full model for the outcome $ACP_{hi}=1$ if an ACP conversation occurred at any point during the patients hospital stay (0 otherwise) has the form:

$$logit\left( \Pr\left( {ACP}_{hi}=1 | \theta_{h} \right) \right)=\beta_{0}+\beta_{1}^{T}PatientRace_{hi}+\beta_{2}^{T}ProviderRace_{hi}+\beta_{3}^{T}RaceInteraction_{hi}+\beta_{4}PatientAge_{hi}+\beta_{5}ProviderAge_{hi}+\beta_{6}PatientSex_{hi}+\beta_{7}ProviderSex_{hi}+\beta_{8}^{T}CharlsonComorbidity_{hi}+\beta_{9}MortScore_{hi}+\beta_{10}SQ_{hi}+\beta_{11}IMG_{hi}+\theta_{h}$$

where $RaceInteraction_{hi}$ is either $ConcordantRace_{hi}$, the 3 binary $ConcordantRaceK_{hi}$ variables or the 9 binary $CombinationRace{KL}_{hi}$ variables that are included in the model along with the patient and provider race main effects and $\theta_{h}\sim Normal\left( 0,\tau^{2} \right)$ is the random-effect for hospital $h$.

To determine which of the three specifications fits the data the best, we use type III analysis of variance type tests to determine if the improved fit of models with more flexible specifications of $RaceInteraction_{hi}$ fit the data statistically significantly better than with those with more parsimonious specifications. That is, we compare the model with:

$$RaceInteraction_{hi}= CombinationRace{KL}_{hi}$$

to the model with

$$RaceInteraction_{hi}= ConcordantRaceK_{hi}$$

to the model with

$$RaceInteraction_{hi}= ConcordantRace_{hi}$$

to the model with no race terms (i.e., $RaceInteraction_{hi}$ excluded). These comparisons form a nested sequence such that the first model contains all the predictors contained in the subsequent model and so must fit the data at least as well as the subsequent model. The statistical test determines whether the larger model fits the data significantly better or not.

**Appendix II**

| **Variable** | **Data Source** | **Time Period** | **Derivation Details** |
| --- | --- | --- | --- |
| Admitting diagnosis | MedPAR | Index stay | Admitting diagnosis code on the MedPAR record was used to create CMS hierarchical condition categories (CMS-HCC) that were included in mortality model individually |
| Admission status | MedPAR, Inpatient, Outpatient | Index stay | Categories derived from admission type code. |
|  |  |  | Elective: 3 (elective) or missing |
|  |  |  | Urgent: 1 (emergency), 2 (urgent), 5 (trauma) or if the patient was admitted through the emergency department (identified by revenue center codes 0450-0459, 0981 on the inpatient or outpatient claim or an emergency room charge amount greater than $0 on the Medpar record) |
| ICU utilization | Inpatient | Index stay | Indicator variable derived from revenue center codes 020X (intensive care) and 021X (coronary care) on the inpatient record |
| Charlson comorbidities | MedPAR, Inpatient, Outpatient, Carrier/Part B, Skilled Nursing Facility, Home Health Agency, Hospice | 1-year lookback | Myocardial infarction, congestive heart failure, peripheral vascular disease, cerebrovascular disease, dementia, chronic respiratory disease, mild liver disease, diabetes with and without complications, hemi/paraplegia, renal disease, non-metastic cancer, moderate to severe liver disease, metastatic cancer and HIV/AIDS (Reference: Quan et al., 2005). |
| Emergency department visits | MedPAR, Inpatient, Outpatient | 1-year lookback | Revenue center codes 0450-0459, 0981 on the inpatient or outpatient claim or an emergency room charge amount greater than $0 on the Medpar record |
| Oxygen use | Durable medical equipment claims | 1-year lookback | HCPCS codes E1390, E1391, E0424, E0431, E0433, E0434, E0439, E1405, E1406, E1392, E0441, E0442, E0443, E0444, K0738 on durable medical equipment claims |
| Urgent readmission status | MedPAR | 30-day lookback | Index stay with an admission date occurring within 30 days after the discharge date on a previous Medpar record in patients with an urgent admission type |
| Transfer status | MedPAR | Index stay | An in hospital transfer was identified by admission source code D and an out of hospital transfer was identified by admission source code 4 on the MedPAR record |
| Dependent on services, residing in a nursing home, on hospice prior to admission | MedPAR, Carrier/Part B, Skilled Nursing Facility, Home Health Agency, Hospice | 30-day lookback | Skilled nursing, home health and hospice dependence were identified by any evidence of utilization within the 30 days prior to admission or if the admission source code on the MedPAR record indicated a transfer from a skilled nursing facility (code 5) or hospice (code F); nursing home care was identified using HCPCS codes 99304-99310, 99315, 99316, 99318 on Part B claims |
| Serious illness | MedPAR, Carrier/Part B | Index stay | Diagnosis fields from the MedPAR record and/or any Part B claims associated with the index stay were used to flag stroke, liver disease, hip fracture, cancer, heart disease, lung disease, neurodegenerative disease, diabetes, HIV/AIDS, renal failure and/or dementia as indicators of serious illness. (Reference: Kelley et al., 2021). |

**Appendix III**

**Logistic regressions of ACP conversations by physician–patient race (balanced random sample: 3,000 patients per race group).**

|  | Model 1 | Model 2 | Model 3 | Model 4 |
| --- | --- | --- | --- | --- |
| Patient Race (White Comparison) |  |  |  |  |
| *Asian* | 0.61*** (0.095) | 0.60*** (0.050) | 0.77* (0.090) | 0.67** (0.078) |
| *Hispanic* | 0.81** (0.082) | 0.82* (0.063) | 0.86 (0.074) | 0.73*** (0.076) |
| *Black* | 0.85* (0.026) | 0.86* (0.064) | 0.91 (0.079) | 0.83 (0.100) |
| Provider Race (White Comparison) |  |  |  |  |
| *Asian* | 1.04 (0.065) | 1.04 (0.065) | 1.15 (0.095) | 0.94 (0.091) |
| *Hispanic* | 0.72* (0.129) | 0.71* (0.128) | 0.86 (0.128) | 0.68 (0.150) |
| *Black* | 0.97 (0.086) | 0.97 (0.086) | 1.05 (0.116) | 0.89 (0.137) |
| Race Interactions |  |  |  |  |
| *Concordance* |  | 1.03 (0.063) |  |  |
| *White Concordance* |  |  | 1.20 (0.135) |  |
| *Asian Concordance* |  |  | 0.81 (0.104) | 1.02 (0.145) |
| *Hispanic Concordance* |  |  | 1.32 (0.301) | 1.54 (0.494) |
| *Black Concordance* |  |  | 0.90 (0.172) | 1.02 (0.232) |
| *Asian Patient * Black Provider* |  |  |  | 1.16 (0.248) |
| *Asian Patient * Hispanic Provider* |  |  |  | 0.95 (0.361) |
| *Hispanic Patient * Asian Provider* |  |  |  | 1.48** (0.220) |
| *Hispanic Patient * Black Provider* |  |  |  | 1.40 (0.347) |
| *Black Patient * Asian Provider* |  |  |  | 1.06 (0.158) |
| *Black Patient * Hispanic Provider* |  |  |  | 0.84 (0.332) |
| Patient Age | 1.01 (0.004) | 1.01 (0.004) | 1.01 (0.004) | 1.01 (0.003) |
| Provider Age | 0.99** (0.003) | 0.99*** (0.003) | 0.99*** (0.003) | 0.99*** (0.003) |
| Patient Female | 1.09 (0.054) | 1.09 (0.054) | 1.09 (0.054) | 1.09 (0.054) |
| Provider Female | 0.94 (0.044) | 0.93 (0.045) | 0.93 (0.045) | 0.93 (0.044) |
| Mortality Model Score | 3.28*** (0.533) | 3.28*** (0.534) | 3.29*** (0.535) | 3.29*** (0.535) |
| Surprise Question | 2.34*** (0.144) | 2.34*** (0.135) | 2.34*** (0.135) | 2.34*** (0.135) |
| International Medical Graduate | 0.66** (0.095) | 0.67** (0.090) | 0.67** (0.090) | 0.67** (0.090) |
| Number of Encounters | 46,218 | 46,218 | 46,218 | 46,218 |
| Model Fit (AIC) | 15474.86 | 15275.78 | 15277.70 | 15281.79 |
